# A protocol of rapid laboratory evolution by genome shuffling in *Kluyveromyces marxianus*

**DOI:** 10.1016/j.mex.2020.101138

**Published:** 2020-11-12

**Authors:** Li Wu, Mengzhu Wang, Genhan Zha, Jungang Zhou, Yao Yu, Hong Lu

**Affiliations:** a State Key Laboratory of Genetic Engineering, School of Life Sciences, Fudan University, Shanghai 200438, China; b Shanghai Engineering Research Center of Industrial Microorganisms, Shanghai 200438, China; c Shanghai Collaborative Innovation Center for Biomanufacturing, East China University of Science and Technology, 130 Meilong Road, Shanghai 200237, China

**Keywords:** Genome shuffling, Mating, Ideal phenotypes

## Abstract

Genome shuffling is a process to combine advantage traits by the recombination of the entire genome and it has been successfully applied in the laboratory evolution of various industrial microorganisms. However, genome shuffling has not been described in *Kluyveromyces marxianus* (KM), a promising yeast host for the expression of heterologous proteins. In this protocol, genome shuffling in KM is performed by sexual reproduction and is combined with high-throughput screening to obtain high-yielding strains. Notably, the screening of diploid clones risen from one mating mixture is carried out to improve the effectiveness of evolution. Mating-sporulation-mating cycles are repeated to obtain KM strain with ideal traits.

• The method combines genome shuffling with high-throughput to achieve strains displaying high yielding of heterologous proteins.
• This method can be applied to the genome shuffling of other species when only a few starting strains are available for sexual reproduction.

## Graphical abstract

**Figure:**
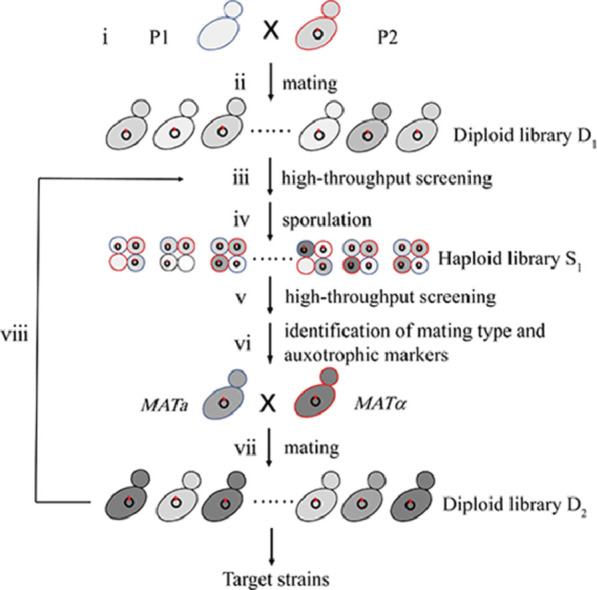

## Specifications table [[1], [2], [3], [4], [5]]

**Table:** 

Subject Area:	Biochemistry, Genetics and Molecular Biology
More specific subject area:	Genetic engineering of yeast
Method name:	Genome shuffling and screening in *Kluyveromyces marxianus*.
Name and reference of original method:	NO
Resource availability:	**Reagents:** YPD solid medium: 10 g/L yeast extract, 20 g/L hipolypepton, 20 g/L glucose, 20 g/L agar ME medium: 50 g/L malt extract, 30 g/L agar SD medium: 6.7 g/L yeast nitrogen base, 10 g/L glucose, 20 g/L agar YPA medium: 20 g/L potassium acetate, 20 g/L bactopeptone, 10 g/L yeast extract 1 × PBST: 8 g/L NaCl, 0.2 g/L KCl, 1.42 g/L Na_2_HPO_4_, 0.27 g/L KH_2_PO_4_, 2.5% Triton-X-100, pH:6.4 SC-Ura medium: 10 g/L glucose, 6.7 g/L yeast nitrogen base, 40 mg/L histidine, 40 mg /L leucine, 40 mg/L tryptophan, 20 g/L agar YG liquid medium: 20 g/L yeast extract, 40 g/L glucose SC-Ura-His-medium: 10 g/L glucose, 6.7 g/L yeast nitrogen base, 40 mg /L leucine, 40 mg/L tryptophan, 20 g/L agar SC-Ura-Trp-medium: 10 g/L glucose, 6.7 g/L yeast nitrogen base, 40 mg/L histidine, 40 mg /L leucine, 20 g/L agar 0.2% SDS Nonidet P-40 2% potassium acetate Zymolyase (5 U/µl, E1004, Zymoresearch, USA) β-Mercaptoethanol (0482, Amresco) chloro‑4-nitrophenyl ferulate (Meilunbio) Nuclease-free water **Materials:** Bioruptor (UCD-300, Crouzet) 96-well microplates (HYK-Z11–2, Haiyinkang) PCR machine (2720, Applied Biosystems) Nuclease-free 1.5 ml Eppendorf tubes Taq DNA Polymerase (E001, Novoprotein)

## Method details

**Fig. 1:**
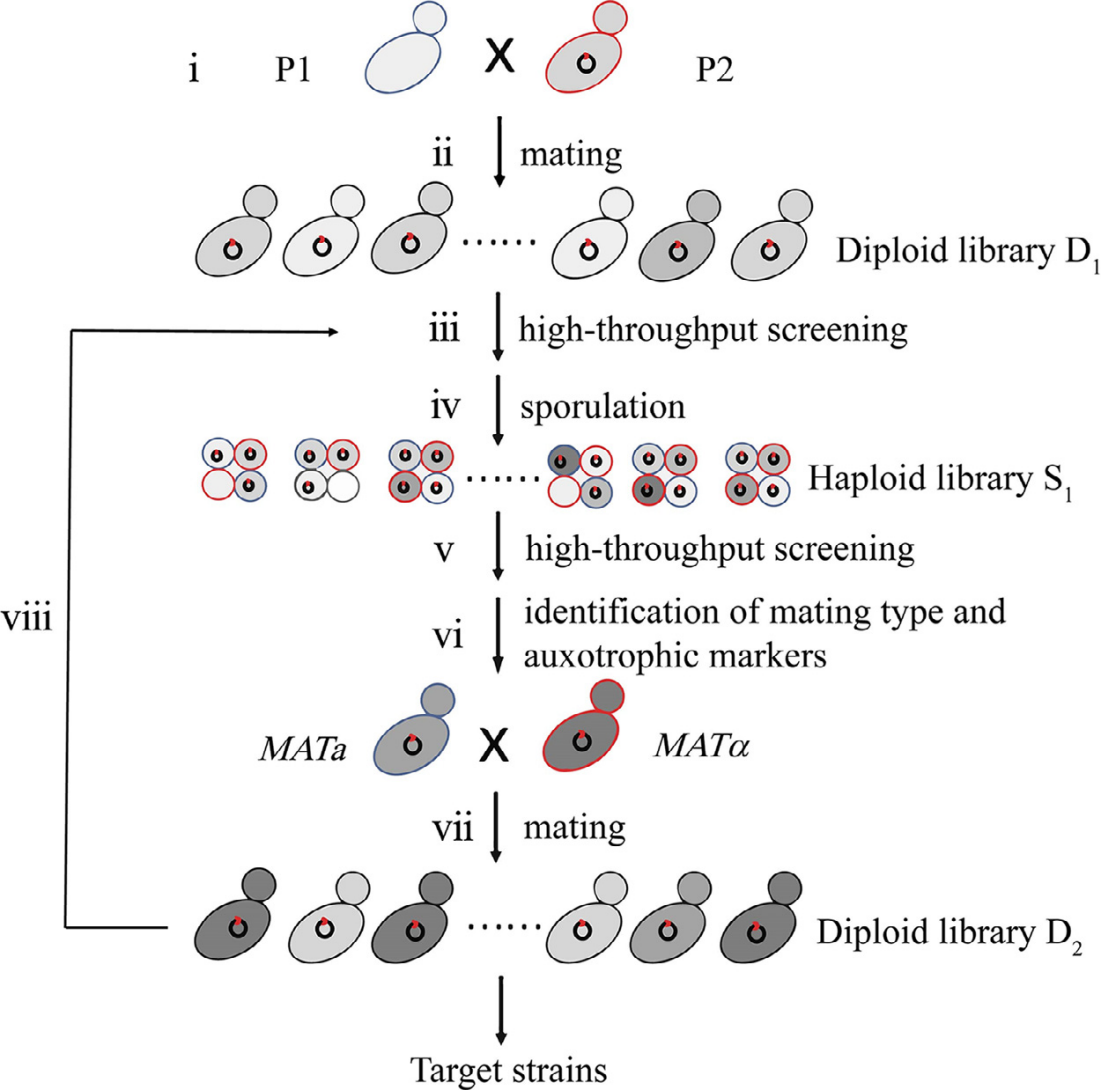
Graphical overview of the genome shuffling and screening in KM. (i) Parental strains with different mating type and auxotrophic markers are constructed (P1:*MAT*a *ura3*Δ*his3*Δ, P2:*MAT*α *ura3*Δ*trp1*Δ). P2 strain contains a plasmid expressing a heterologous protein. (ii) Parental strains mate and diploid clones from the mating mixture form the first diploid library D_1_. (iii) (iv) Diploid clones of D_1_ are grown in 96-well microplates and the best strain that displaying the highest yielding of heterologous protein is selected by high-throughput screening, which is subjected to the sporulation in a medium containing limited nutrients and spores form the first haploid library S_1_. (v) Haploid clones of S_1_ are subjected to the high-throughput screening as diploid clones. (vi) Haploid clones displaying a high level of yielding are subjected to the identifications of mating type and auxotrophic markers. (vii) Haploid cells with compatible mating types and auxotrophic markers are selected for mating. Diploid clones from the mating mixture formed the second diploid library D_2_. (viii) Diploid clones of D_2_ are subjected to the high-throughput screening. Selected diploid can go through the cycle until KM strains with ideal traits are obtained.

*Kluyveromyces marxianus* (KM) has two mating types designated as *MAT*a and *MAT*α [1]. Upon harsh environments, haploid cells of opposite type mate to produce diploid and form spores. In KM haploid cells, the mating-type switch happens and the frequency of switching varies depending on the strains. In this protocol, genome shuffling in KM is performed by recursive mating, which can circumvent the low efficiency of protoplast fusion [2]. In order to improve the efficiency of genome recombination, parent strains have different genetic backgrounds.The feruloyl esterase Est1E is used as a marker protein to measure the capacity of KM to express a heterologous protein. The expression level of Est1E is detected by a color-based assay [3]. The *HML* or *HMR* locus in the original parental strain is removed to produce stable haploid strain. Haploid cells carry auxotrophic markers to facilitate the selection of diploid. Considering the genetic diversity in diploid cells due to mitotic recombination, we perform a high-throughput screening of diploid clones from one mating mixture to improve the effectiveness of the selection. Diploid cells sporulated and spores are screened by the same method. The mating types and auxotrophic markers of the spores displaying improved features are identified to find pairs for the next round of mating. Mating-sporulation-mating cycle can be repeated until KM strains with ideal phenotypes are obtained (Fig. 1).

### Construction of parental strains

The parental strain used in this study is originated from FIM-1 through UV-^60^Co-γ irradiation method. The two parental stains all have hundreds of specific mutations that not isogenic. To obtain a stable haploid parental strain, the *HML* locus in a *MAT*a strain and *HMR* locus in a *MAT*α strain is deleted. This step is not necessary for some KM strains that seldom switch mating type. To provide auxotrophic markers for the selection of diploid, *HIS3* in the *MAT*a strain and *TRP1* in the *MAT*α strain is deleted. Both parental strains contain a deletion of *URA3* to be compatible for a *URA3* plasmid expressing the heterologous protein. The deletion is performed by the recombination with the aid of a CRISPR plasmid as described before [3].

### Mating

1. *MAT*a and *MAT*α cells are grown separately on the YPD solid medium at 30 °C for about 12 h. Inoculate the cells by patching to produce enough cells for the next step.
2. Similar to *K. lactis*, the mating of KM happens in the medium containing limited nutrients [4]. Scrape off two matchheads of *MAT*a and *MAT*α cells from YPD plates and mix them thoroughly on the ME medium by toothpicks. Spread out the mixture to make a thin layer above the medium. Culture the ME medium plates at 30 °C for 2 days.
3. Scrape off four matchheads of cells from the ME medium and transfer them to a 1.5 ml Eppendorf tube by toothpicks. Serial dilute the cells by sterile water and spread them onto SD medium plate to select for diploid cells. Usually, 100 µl 10^−^^5^ dilution produces a few hundred well-separated clones on the SD medium.
4. Inoculate clones in SD medium and grow at 30 °C for 1 day.

### High-throughput screening

The purpose of this step is to select the diploid or haploid clones with ideal traits. High-throughput screening is performed in 96-well microplates. Selection of clones yielding high-level of Est1E is described in this protocol. The protocol can be modified to screen clones expressing other heterologous proteins or clones with other ideal traits.

1. Inoculate single clone into a well of 96-well microplate containing 600 µl YG liquid medium. Culture the cells at 30 °C for 72 h.
2. Centrifuge the 96-well microplate at 4000 rpm for 10 min. Transfer 20 µl supernatant to a new 96-well microplate. Add 170 µl 1 × PBST and 10 µl 20 mM 2‑chloro-4-nitrophenyl ferulate. Mix thoroughly.
3. Incubate the mixture at 30 °C for 20 min. The activity of Est1E is indicated by the absorbance at a wavelength of 410 nm.
4. To validate the clones, inoculate clones yielding high activity of Est1E into 50 ml YG liquid medium. Culture the cells at 30 °C for 72 h with agitation. Measure the enzymatic activity of Est1E in the supernatant as described above. Clones that have passed the validation are ready for the next step.

### Sporulation

1. Inoculate the diploid cells into 3 ml YPD liquid medium and culture at 30 °C for 12 h.
2. Inoculate the cultures into 50 ml of YPA-medium to start at an optical density at 600 (OD_600_) of 0.1. Culture the cells at 30 °C for 6–8 h until the OD_600_ reach 1.0–1.2 (~2 × 10^7^ cells/ml). Careful monitoring of OD_600_ is required to achieve efficient sporulation.
3. Transfer all the culture into a 50-ml sterile tube and centrifuge at 5000 g for 5 min. Discard the supernatant and resuspend the cells in 10 ml sterile H_2_O. Centrifuge at 5000 g for 1 min and discard the supernatant.
4. Resuspend the cells in 50 ml 2% potassium acetate and incubate the cells at 30 °C with agitation.
5. Monitor the sporulation by microscopic observation. Usually, tetrads are observable in 12 h and most spores are released from the asci after 72 h.
6. Add 1 ml cultures into a 1.5 ml Eppendorf tube and centrifuge at 8000 rpm for 1 min. Discard the supernatant and wash the cells twice by 1 ml sterile water.
7. Resuspend the cells in 500 µl sterile H_2_O supplemented 25 µl of Zymolyase and 5 µl of β-mercaptoethanol. Incubate the mixture at 30 °C for at least 12 h. Zymolyase is used to digest vegetative cells and asci.
8. Add 200 µl of 1.5% Nonidet P-40 (NP-40) into the suspension. Mix thoroughly and incubate at 30 °C with shaking for 30 min at least. This step kills residual protoplastic cells.
9. Sonicate the cells at high frequency for 30 s for 6 times to disperse tetrads and clumps. Check the dispersion of spores under a microscope and sonicate more if necessary.
10. Centrifuge at 8000 rpm for 10 min. Discard the supernatant and resuspend the cells in 500 µl sterile H_2_O. Serial dilute the spores by sterile H_2_O and spread onto SC-Ura medium. Incubated at 30 °C for 2–3 days until colonies are visible. Usually 100 µl 10^−5^ dilution produces a few hundred clones on the medium. Haploid clones are ready for the high-throughput screen as described above.

### Identification of spores

Verify the mating type of haploids by PCR using Taq DNA polymerase. Scrape off a matchhead of cells into 30 µl 0.2% SDS and mixed them thoroughly. Boil the sample for 5 min in boiling water. Centrifuge the sample at 13.2 k rpm for 1 min and the supernatant is used as a template for PCR. *MAT*a locus produces a band of 1062 bp by the primer pair of YY270F (5′ TGCAACCAAC CAATCCCTTCCAAATTC 3′) and YY271F (5′ TCTTCCTTGA ACCCGAAGCAAAAGATC 3′). *MAT*α locus produces a band of 1515 bp by using primer pair of YY270F and YY272F (5′ AACTTCAATC CCCGACCCACCGCAGTC 3′). Identify the auxotrophic markers by inoculating haploids onto SC-Ura-His-medium and SC-Ura-Trp-medium. Incubate haploids at 30 °C for 24 h.

## Method validation

This method was successfully applied to improve the yielding of a heterologous protein in KM. After two rounds of mating and screening, a diploid strain called D_2–13_ was obtained which displayed a 5-fold increase of secretory activity of Est1E compared to the parental strains [5]. Although only two parental strains were selected for mating, the method included a screening of diploid clones from one mating mixture to improve the effectiveness of evolution. Therefore, this method is useful for the genome shuffling of other species when only a few starting strains are available for sexual reproduction.

## Acknowledgments

This work was supported by the 10.13039/501100001809National Natural Science Foundation of China [Grant Numbers 31770094, 31970068 and 31970549], the National High Technology Research and Development Program of China [Grant Numbers 2014AA021301], the Science and Technology Research Program of Shanghai [Grant Number 19395800600, 18391901800, 19DZ2282100] and the Open Research Funds of the State Key Laboratory of Genetic Engineering, Fudan University.

## Declaration of Competing Interests

The authors declare that they have no known competing financial interests or personal relationships that could have appeared to influence the work reported in this paper.

## Supplementary material and/or additional information

None.
